# Clinical Reports—Veterinary Hospital, University of Pennsylvania

**Published:** 1898-03

**Authors:** S. J. J. Harger


### CLINICAL REPORTS—VETERINARY HOSPITAL, UNIVERSITY OF PENNSYLVANIA.

CLINIC OF S. J. J. HARGER, V.M.D.

CASE I. *Pruriginous erythema, a trophic sequel to plantar-neurectomy.*—Patient, bay gelding. Was purchased as sound for a high price. Last June showed slight lameness in the off forefoot.

I was called to treat the animal for an intense pruritis of the pastern, the animal constantly endeavoring to bite the parts. A previous neurectomy was suspected, and, after some difficulty, the cicatrices of the high-plantar operation were found; also small neuromas.

In order to relieve the lameness, the same operation was again performed. In about a month the itching of the pastern and coronet, which had disappeared after the operation, reappeared. The pruritis was excessive, and the patient constantly endeavored to bite the skin or rub it with the opposite foot, or against the manger, even leaving his feed to obtain relief in this manner. The skin became depilated over an area larger than a silver dollar, red (white foot), somewhat tumefied, and was covered with a moist, serous exudate; no vesicles or sloughing of the integument.

*Treatment.* The lesion was very obstinate to treatment. The animal had to be constantly "tied up" in the stall, and watched while eating. The skin was cleansed daily and sprinkled with an antiseptic mixture—iodoform, tannic acid, and charcoal. The diseased parts were protected from the frantic desire of the animal's biting by a leather ankle-boot extending to the hoof and covering the part. Two months were required for a recovery, and no trace of the trouble is left.

*Remarks.* This condition may follow median as well as high-plantar neurectomy; its seat is always the same. There may exist a number of these patches. They may assume an eczematous appearance, and may be scaly or covered with vesicles and pustules partly due to traumatisms by the animal. This trophic alteration is seen very rarely. In about fifty or, perhaps, more cases the writer has witnessed it only once, although he has seen a second case in the practice of a colleague.

Its principal characteristics are its infrequency, its obstinacy to treatment, and its being premonitory of a previous nerve resection. Its existence *always* demands a most scrutinous search for the *scars of a neurectomy*. It is not indicative of suppuration of the foot, but apparently always heals after a variable period.

CASE II. *Oöphorectomy in a mare.*—Patient a bay mare, driver, six years old. Sold a year before, on account of her disposition. Appetite poor, condition emaciated. The animal was constantly in heat, irritable, "switched" the tail and kicked, both when approached in the stall and when in harness; was unsafe as a driver, and practically unserviceable. Usual symptoms of nymphomania.

Oöphorectomy was performed last March, the animal standing, after administering an ounce of chloral, and after ten days at the hospital the patient was discharged. The ovaries were removed in the usual way through the vagina. Instead of the écraseur, the instrument devised by Dr. F. F. Hoffman, of Brookville, Pa., was used; instead of having a chain, this instrument has a movable rod with a cutting-hook at the free end. The pedicle of the ovary can be more easily secured than with the écraseur.

The left ovary was normal; the right was the seat of a cyst about as large as a walnut.

After the operation the mare for some time remained much emaciated, but gradually recovered; for about a week in a month she would menstruate and show some of her old habits in a mild form. These symptoms, however, gradually disappeared until they are not manifest in the stable or in harness, and the animal can be driven with safety.

CASE III. *Extirpation of the parotid gland.*—The patient was a gray gelding, sixteen years old. When purchased by the owner, a little over two years ago, there was a small tumor over the region of the left parotid gland. This growth continued to increase in volume until the entire area of the gland was involved. The animal at times showed symptoms of what the owner called "staggers;" one attack, in particular, occurred during early summer after a sudden change in the temperature to a few hot days, and continued for several days. It probably was passive congestion of the brain, from parotid compression of the jugular vein.

The owner concluded to have the animal placed under treatment, and, by the advice of Dr. H. D. Hackler, sent him to the hospital last May.

The animal was placed on the operating-table, and the entire removal of the gland was deemed necessary. A longitudinal incision involving the skin, the cuticularis colli, and the parotid auricularis muscles was made from the base of the ear to the lower extremity of the gland. Owing to the fact that the entire removal of the parotid gland has been seldom, if ever, accomplished, at least as far as I am aware of, and its close relationship with the jugular vein, which passes through the gland, the maxillo-muscular, posterior auricular, and the external carotid arteries, which lie on its deep face and supply it with large branches, and finally the seventh pair of cranial nerves, the motor nerve of the facial muscles, the utmost care was necessary in enucleating the gland and its tumor. The connective tissue was separated from the surface of the gland with the fingers, and when this was not possible the curved scissors was employed. The jugular vein, and the arterial branches were ligated as they were met. The hemorrhage was quite copious. The animal was somewhat emaciated, although he seemed none the worse from loss of blood after the operation. After the hemorrhage was arrested and the parts cleansed a perforated rubber drainage-tube, with the ends protruding from each commissure of the incision, was inserted and the lips of the wound united with interrupted sutures. The usual antiseptic treatment was given.

After the operation the lower lip on the left side was pendulous and the movements of the eyelids and the ear were much diminished, due to injury to the facial nerve. The wound granulated kindly, and the animal was discharged in fourteen days.

*Present condition.* At present writing there is no deformity of the parotid region, and no cicatrix apparent on casual observation. The movements of the eyelids and ear are normal; the lips are normal, excepting a slight deviation toward the right side; never any interference with prehension of food. The jugular vein, posterior to the gland, is not obliterated; this was prevented by the current of blood from the glosso-facial and occipital veins. General condition excellent and no unfavorable sequela. The tumor, as was suspected, proved to be a melanotic sarcoma.

---

The law prohibiting the importation of diseased cattle into Pennsylvania, with the proclamation, has just been issued by the board.

